# Polymicrobial methicillin-resistant *Staphylococcus aureus* bloodstream infections

**DOI:** 10.1128/spectrum.01081-24

**Published:** 2024-09-25

**Authors:** Angelica Escalona, Emi Hayashi, Michelle Evans, Harm van Bakel, Bremy Alburquerque, Amy C. Dupper, Russell McBride, Deena R. Altman

**Affiliations:** 1 Department of Medicine, Division of Infectious Diseases, Icahn School of Medicine at Mount Sinai, New York, New York, USA; 2 Department of Genetics and Genomics Sciences, Icahn School of Medicine at Mount Sinai, New York, New York, USA; 3 Department of Microbiology, Icahn School of Medicine at Mount Sinai, New York, New York, USA; 4 Icahn Genomics Institute, Icahn School of Medicine at Mount Sinai, New York, New York, USA; 5 Department of Artificial Intelligence And Human Health, Icahn School of Medicine at Mount Sinai, New York, New York, USA; 6 Department of Pathology, Molecular and Cell Based Medicine, Icahn School of Medicine at Mount Sinai, New York, New York, USA; University of California, San Diego, La Jolla, California, USA

**Keywords:** *Staphylococcus aureus*, bloodstream infections, bacteremia

## Abstract

Methicillin-resistant *Staphylococcus aureus* (MRSA) bloodstream infection (BSI) is a serious public health concern. At times, MRSA is isolated from the blood along with other pathogens, the significance and consequences of which are not well described. This study aims to outline the clinical characteristics and outcomes of those with polymicrobial MRSA BSI compared with those with monomicrobial MRSA BSI. We conducted a retrospective case-control study of those with and without polymicrobial MRSA BSI from 2014 to 2022 at a single quaternary care center in New York City. Risk factors and outcomes for polymicrobial MRSA BSI were assessed using logistic regression analyses. Of 559 patients with MRSA BSI during the study period, 49 (9%) had polymicrobial MRSA BSI. Gram-positive *Enterococcus* (23%) was the most common co-pathogen. The presence of urinary (*P* = 0.02) and gastrointestinal (*P* < 0.01) devices was significantly associated with polymicrobial MRSA BSI. Polymicrobial MRSA BSI was associated with intensive care unit (ICU) admission after BSI (*P* = 0.01). Mortality did not differ. While polymicrobial MRSA BSI is relatively uncommon, it complicates an already complex clinical scenario of MRSA BSI.

**IMPORTANCE:**

*Staphylococcus aureus* is a common human pathogen associated with severe disease and high mortality rates. Although clinically observed, little is known about the impact of polymicrobial staphylococcal bloodstream infection. This study evaluates polymicrobial methicillin-resistant *S. aureus* bloodstream infection (BSI), highlighting the increased risk of intensive care unit admission and impact on morbidity. Identifying risk factors for polymicrobial BSI, such as the presence of specific devices, can aid in early recognition and targeted interventions. Clarifying the risks and outcomes of polymicrobial infections can lead to strategies to minimize and manage these infections and explore the potential interactions between pathogens.

## INTRODUCTION

*Staphylococcus aureus* is a common pathogen that frequently causes bloodstream infections (BSI) in community and hospital settings (1). The overuse and misuse of antibiotics have led to the global emergence of methicillin-resistant *Staphylococcus aureus* (MRSA), which is associated with poor outcomes, including a mortality rate of up to 30% (2). Although most BSI are caused by a single pathogen, polymicrobial BSI occurs when more than one species of pathogen is found in the blood culture. The general incidence of polymicrobial BSI ranges from 6% to 11% (3, 4). Polymicrobial BSI has been associated with worse outcomes when compared to monomicrobial BSI (5). These outcomes have been attributed to an increased likelihood of septic shock, prolonged stays in the intensive care unit and hospital, as well as higher mortality (5). In addition, there is growing awareness of the impact of microbial interactions, and defining the potential of these pathogen interactions in disease severity and outcome may lead to innovative preventative and therapeutic measures for combating infection (6).

Only a small number of studies have focused on clinical characteristics and outcomes of *Staphylococcus aureus* in the setting of polymicrobial BSI (5, 7). We hypothesized that several risk factors may be associated with polymicrobial MRSA BSI, particularly the source of infection, and presence of invasive devices and that polymicrobial infections may result in worse outcomes. This study aims to outline the clinical characteristics and outcomes of those patients with MRSA polymicrobial BSI and compare them with those with monomicrobial MRSA BSI.

## RESULTS

**Fig 1:**
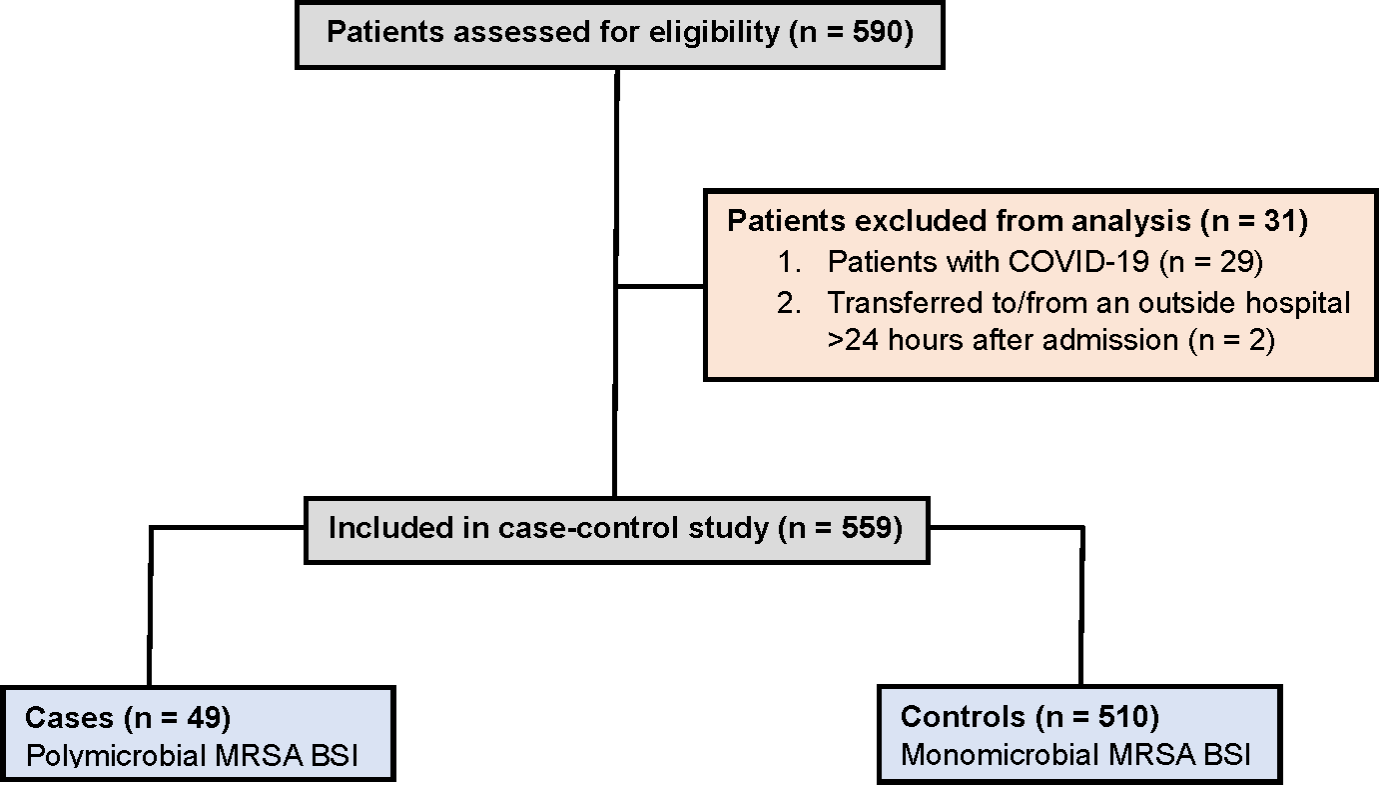
Flowchat outlining the selection of cases and controls with predefined inclusion and exclusion criteria.

During the 8-year study, 559 adult patients had at least one positive blood culture for MRSA, of which 49 (9%) had polymicrobial MRSA BSI (cases) (Fig. 1). In order to mitigate potential confounding effects attributed to coronavirus disease 2019 (COVID-19), 29 patients with severe acute respiratory syndrome coronavirus 2 (SARS-CoV-2), who required treatment directed at SARS-CoV-2 during the same admission for MRSA BSI, had a positive SARS-CoV-2 test within 2 weeks prior to or after the BSI diagnosis, or were symptomatic due to SARS-CoV-2 per clinical documentation were excluded. Demographics and clinical characteristics of cases of polymicrobial and controls with monomicrobial MRSA BSI are summarized in Table 1. The majority of patients were males (63%), and aged 55 and above. Skin infections were the main source of MRSA BSI, representing 27% of polymicrobial cases and 30% of monomicrobial cases. Infections related to vascular access constituted 29% of polymicrobial and 25% of monomicrobial cases. In the study, 21 patients (43%) with polymicrobial MRSA BSI had a central line, compared to 185 patients (36%) in the control group. There was no significant association found between the presence of a central line and polymicrobial MRSA BSI. The average Charlson Comorbidity Index (CCI) (8) among patients was 5.7.

**TABLE 1:** Demographics, clinical characteristics, and univariate analysis of polymicrobial MRSA BSI^*l*^

Characteristic, *n* (%)	Cases (*n* = 49)	Controls (*n* = 510)	OR (95% interval)	*P* value
Sex				
Male	37 (76)	313 (61)	1.94 (0.99–3.81)	0.05
Female	12 (24)	197 (39)	Reference	
Age at time of infection				
18–54 years	13 (27)	162 (32)	Reference	
55–69 years	18 (37)	161 (32)	1.39 (0.66–2.94)	0.44
≥70 years	18 (37)	187 (37)	1.99 (0.57–2.52)	0.96
Race/ethnicity				
Non-Hispanic White	19 (39)	176 (35)	Reference	
Non-Hispanic Black	14 (29)	136 (27)	0.95 (0.46–1.97)	0.90
Hispanic	9 (18)	117 (24)	0.71 (0.33–1.51)	0.97
Other	15 (31)	154 (30)	1.02 (0.54–1.93)	0.95
Missing/unknown	1 (2)	27 (5)		
History of IV drug use				
Yes	6 (12)	61 (12)	1.03 (0.42–2.51)	0.95
No	43 (88)	449 (88)	Reference	
Body mass index (BMI)				
<18.5	5 (10)	57 (11)	0.74 (0.28–1.95)	0.80
18.5–29.9	37 (76)	310 (61)	Reference	
≥30.0	7 (14)	141 (28)	0.42 (0.18–0.96)	0.12
HIV diagnosis				
Yes	4 (8)	42 (8)	0.99 (0.34–2.89)	0.99
No	45 (92)	468 (92)	Reference	
Admission source				
Home^*a*^	27 (55)	308 (61)	Reference	
NH/rehab/LTACH	10 (20)	65 (13)	1.76 (0.81–3.80)	0.15
Outside hospital	12 (24)	136 (27)	1.01 (0.50–2.05)	0.45
Prior hospital admission (90 days)				
Yes	31 (63)	327 (64)	1.04 (0.57–1.91)	0.91
No	18 (37)	183 (36)	Reference	
Presence of invasive devices				
Yes	42 (86)	360 (71)	2.5 (1.10–5.69)	0.03
No	7 (14)	150 (29)	Reference	
Invasive device				
Cardiac^*b*^	8 (16)	53 (10)	1.68 (0.75–3.78)	0.21
Vascular^*c*^	22 (45)	227 (45)	1.02 (0.56–1.83)	0.96
Orthopedic	5 (10)	58 (11)	0.89 (0.34–2.32)	0.81
Urinary^*d*^	21 (43)	135 (26)	2.08 (1.14–3.79)	0.02
Gastrointestinal^*e*^	15 (31)	73 (14)	2.64 (1.37–5.09)	<0.01
Other^*f*^	6 (12)	31 (6)	2.16 (0.85–5.46)	0.10
Clonal complex (CC)				
CC5	23 (47)	209 (41)	Reference	
CC8	14 (29)	206 (40)	0.62 (0.31–1.23)	0.17
Other	11 (22)	55 (11)		
Missing	1 (2)	40 (8)		
Length of hospital stay prior to BSI				
BSI ≤ 3 days after admission	28 (57)	353 (69)	Reference	
BSI > 3 days after admission	21 (43)	157 (31)	1.69 (0.93–3.06)	0.09
Wound present^*g*^				
Yes	31 (63)	299 (59)	1.22 (0.66–2.23)	0.53
No	18 (37)	211 (41)	Reference	
Hemodialysis				
Yes	4 (8)	96 (19)	2.61 (0.92–7.46)	0.07
No	45 (92)	414 (81)	Reference	
Source of infection				
Skin^*h*^	13 (27)	153 (30)	0.84 (0.44–1.63)	0.61
Pneumonia	6 (12)	60 (12)	1.05 (0.43–2.56)	0.92
Bone^*i*^	4 (8)	56 (11)	0.72 (0.25–2.08)	0.55
Vascular^*j*^	14 (29)	130 (25)	1.17 (0.61–2.24)	0.64
Other^*k*^	15 (31)	142 (28)	1.14 (0.60–2.16)	0.68
Charlson comorbidity index				
0–3	15 (31)	141 (28)	Reference	
4–5	6 (12)	110 (22)	0.51 (0.19–1.37)	0.13
6–8	17 (35)	158 (31)	1.01 (0.49–2.10)	0.48
>8	11 (22)	101 (20)	1.02 (0.45–2.32)	0.51

^*a*^ “Home”: included nonmedical residences such as home, group homes, assisted living facilities, and homeless shelters.

^*b*^ “Cardiac (invasive device)”: included pacemakers, implantable cardioverter defibrillators (ICD), left ventricular-assist devices (LVAD).

^*c*^ “Vascular (invasive device)”: included arteriovenous (AV) grafts and other vascular invasive devices.

^*d*^ “Urinary (invasive device)”: included urinary collection devices (ileal conduits, nephrostomy tubes, suprapubic catheters), and foley catheters.

^*e*^ “Gastrointestinal (invasive device)”: included percutaneous endoscopic gastrostomy (PEG) tubes and ostomies.

^*f*^ “Other (invasive device)”: included chest tubes, wound devices, drains, nasogastric tubes, shunts, tracheostomy, endovascular valves, and stents.

^*g*^ “Wound present”: the presence of a chronic skin wound overlying the sacrum, limb, abdomen, or other body parts.

^*h*^ “Skin (source of infection)”: MRSA infection from peripheral IV, surgical site infection, sacral wound, and midline catheter.

^*i*^ “Bone (source of infection)”: MRSA infection from diabetic foot ulcer and osteomyelitis.

^*j*^ “Vascular (source of infection)”: vascular access devices include a non-tunneled central venous catheter, tunneled catheter (hickman or permacath), implanted port, peripherally inserted central catheter (PICC line), and arteriovenous graft (AVG) and fistula (AVF).

^*k*^ “Other (source of infection)”: MRSA infection from a spinal infection, septic arthritis or cardiac device infection, intra-abdominal infection, GU, or an unknown/not reported source.

^*l*^ Abbreviations: BSI, bloodstream infection; MRSA, methicillin-resistant *Staphylococcus aureus*; HACO, healthcare associated community-onset; ICU, intensive care unit; HIV, human immunodeficiency virus; LTACH, long-term acute care hospital; AIDS, acquired immunodeficiency syndrome.

In the univariate analysis (Table 1), the presence of invasive devices demonstrated a significant association with polymicrobial MRSA BSI (odds ratio [OR], 2.5; 95% confidence interval [CI], 1.10–5.69; *P* = 0.03). Specifically, urinary devices (OR, 2.08; 95% CI, 1.14–3.79; *P* = 0.02) and gastrointestinal devices (OR, 2.64; 95% CI, 1.37–5.09; *P* < 0.01) were associated with polymicrobial MRSA BSI. Cases were found to have a higher prevalence of MRSA BSI with the clonal complex (CC) 5 compared to the CC8 (47% vs 29%) though 41 patients (7%) had incomplete gene typing data and were, therefore, excluded from the typing analysis. Baseline hemodialysis (OR, 2.61; 95% CI, 0.92–7.46; *P* = 0.07) exhibited a *P*-value approaching significance (Table 1).

Polymicrobial MRSA BSI was associated with poorer outcomes, with increased intensive care unit (ICU) admissions after polymicrobial BSI (OR, 2.26; 95% CI, 1.21–4.24; *P* = 0.01) (Table 2). Admission to the ICU before bacteremia was not associated with the subsequent development of polymicrobial MRSA BSI. No confounding effect was observed between ICU admission after BSI and the presence of invasive devices. There were no significant differences in 30-day (OR, 0.82; 95% CI, 0.40–1.69; *P* = 0.98), 60-day (OR, 1.04; 95% CI, 0.51–2,11; *P* = 0.91), and 90-day mortality (OR, 1.07; 95% CI, 0.52–2.18; *P* = 0.86).

**TABLE 2:** Univariate analysis of patient outcomes with polymicrobial MRSA BSI^*c*^

Characteristic, *n* (%)	Cases (*n* = 49)	Controls (*n* = 510)	OR (95% interval)	*P* value
90-day Mortality				
Yes	11 (25)	113 (24)	1.07 (0.52–2.18)	0.86
No	33 (75)	361 (76)	Reference	
Mortality related to MRSA				
Yes	13 (72)	103 (63)	0.66 (0.22–1.94)	0.45
No	5 (28)	60 (37)	Reference	
Recurrent bacteremia^*a*^				
Yes	2 (4)	63 (12)	0.30 (0.07–1.26)	0.98
No	47 (96)	443 (87)	Reference	
Not reported	0 (0)	4 (1)		
Metastatic infection^*b*^				
Yes	9 (18)	99 (19)	0.92 (0.43–1.97)	0.98
No	40 (82)	406 (80)	Reference	
Not reported	0 (0)	5 (1)		
ICU admission after BSI				
Yes	17 (35)	97 (19)	2.26 (1.21–4.24)	0.01
No	32 (65)	413 (81)	Reference	
Intubated after BSI				
Yes	6 (12)	71 (14)	0.86 (0.35–2.10)	0.75
No	43 (88)	439 (86)	Reference	

^*a*^ “Recurrent bacteremia”: MRSA BSI with a positive blood culture over 30 days after the last positive blood culture.

^*b*^ “Metastatic infection”: distal or secondary infection, anatomically unrelated to the primary site of infection, presenting during index admission of MRSA BSI. Includes infective endocarditis, septic pulmonary emboli, vertebral osteomyelitis, septic arthritis, iliopsoas abscess.

^*c*^ Abbreviations: MRSA, methicillin-resistant *Staphylococcus aureus*; BSI, bloodstream infection; ICU, intensive care unit.

We evaluated the types of organisms in polymicrobial MRSA BSI, with Table 3 presenting the frequency of microorganisms identified. *Enterococcus* species was the most frequently detected pathogen, accounting for 23% of cases. Upon further breakdown, *Enterococcus faecalis*, *Enterococcus faecium,* and *Enterococcus avium* were responsible for 18.3%, 3.3%, and 1.6% of the cases, respectively. *Klebsiella*, *Proteus*, and *Streptococcus* species were also present in 11.7% of cases. Most co-pathogens were gram-positive bacteria with a prevalence of 53%, followed by gram-negative at 43%, and yeast at 3% (Table 3).

**TABLE 3:** Frequency of organisms classified by gram stain in polymicrobial MRSA BSI

Organism	Frequency	Percent (%)
Gram positive	32	53
*Bacillus* spp.	2	3.3
*Enterococcus* spp.	14	23.3
MSSA	3	5.0
*Rhodococcus* spp.	1	1.7
*Staph* coag neg spp.	5	8.3
*Streptococcus* spp.	7	11.7
Gram negative	26	43
*Acinetobacter* spp.	2	3.3
*Enterobacter* spp.	1	1.7
*Escherichia* spp.	5	8.3
*Klebsiella* spp.	7	11.7
*Proteus* spp.	7	11.7
*Providencia* spp.	1	1.7
*Pseudomonas* spp.	3	5.0
Yeast	2	3
*Candida* spp.	2	3.3

## DISCUSSION

Bloodstream infection caused by *Staphylococcus aureus* is a serious health concern associated with high morbidity and mortality. Despite its occurrence in the clinical setting, little is known about the implications of polymicrobial MRSA BSI syndrome. In this retrospective case-control study, we found polymicrobial BSI to occur in 9% of all MRSA BSI cases and determined the clinical characteristics and outcomes of those with polymicrobial MRSA BSI. The presence of urinary and gastrointestinal invasive devices was significantly associated with the development of polymicrobial MRSA BSI. A significant association was also found between polymicrobial MRSA BSI and ICU admission after BSI, and the most frequent co-pathogen was gram-positive *Enterococcus*.

Invasive devices in the genitourinary (GU) and gastrointestinal (GI) systems were associated with polymicrobial MRSA BSI. GU devices, such as urinary catheters, can promote the formation of biofilms, enabling the adherence and colonization of microorganisms (9). Similarly, GI devices such as percutaneous endoscopic gastrostomy (PEG) tubes can act as pathways for microorganisms from the external environment, leading to biofilm formation and subsequent complications (6). Hence, the presence of biofilms on GU and GI devices may contribute to the development of polymicrobial MRSA BSI. Moreover, *S. aureus* is well equipped with a host of virulence factors that promote invasion and adherence, such as biofilm formation (1). It is important to note that this study did not establish significant associations between the source of infection and polymicrobial MRSA BSI.

Patients with invasive devices likely have underlying medical conditions that weaken their immune system, making them more susceptible to infection. Healthcare-associated risk factors were prevalent among patients with polymicrobial MRSA BSI. Indeed, this was found in a previous study of polymicrobial bacteremia which demonstrated a correlation between polymicrobial episodes and hospital-acquired infections (10). As devices of the GU and GI tracts are frequently indicative of underlying comorbidities due to their necessity in these comorbidities, this potentially contributes to the observed findings. Our study revealed an average CCI of 5.7 among all patients, exceeding the commonly reported range of 1.5–3 in other studies (5, 8).

In addition to the link between polymicrobial MRSA BSI and healthcare-associated risk factors, the prevalence of specific MRSA clones offers further insights. Our study revealed a higher prevalence of CC5 compared to CC8 genotypes in cases of polymicrobial MRSA BSI. CC5 is historically associated with older individuals with hospital or long-term care facility exposure, whereas CC8 (specifically the USA300 pulsotype) is associated with community settings although studies have found these historical connections are fading (2, 11). Our findings suggest that hospital-adapted clones may lead to the development of polymicrobial MRSA BSI within the hospital setting. Despite the analysis not being able to establish a significant association between polymicrobial MRSA BSI and hospital-onset (HO) MRSA infections, it is crucial to acknowledge that cases of BSI occurring within the first 3 days after admission (community-onset or CO) included mainly those with healthcare-associated community-onset MRSA infection, defined as a positive blood culture within the initial 72 h of admission, but also containing risk factors associated with hospital settings (frequent healthcare interactions, hemodialysis status, hospitalization within the prior 3 months, or residence in long-term acute care facilities). Only three cases of polymicrobial MRSA BSI were identified as truly CO. Characterization of the epidemiology and pathogenesis of clones associated with infections especially in those with invasive devices signal potential areas of intervention in infection control.

We describe an increase in ICU admissions after BSI in polymicrobial MRSA BSI as compared to controls. Infection with multiple pathogens may lead to severe symptoms, complications, and additional therapeutic challenges. The presence of polymicrobial MRSA BSI may also be a marker of underlying comorbidity inherently leading to worse outcomes and the need for ICU care. It is notable that mortality did not differ between the groups. Polymicrobial bacteremia likely increases antibiotic class exposures and may have downstream impacts on the development of microbial drug resistance.

*Enterococcus* spp. was the most common co-pathogen in polymicrobial MRSA BSI, while a previously published study found *Acinetobacter baummanii* predominant co-pathogen, followed by *Enterococcus* spp. (5). *Staphylococcus aureus* and *Enterococcus* spp. are gram-positive pathogens that cause bacteremia and infective endocarditis. While studies have focused on the mechanisms by which *S. aureus* acquires the vancomycin resistance gene (vanA, for example) from vancomycin-resistant *Enterococcus*, no other known synergistic or antagonistic interactions exist between *Enterococcus* spp. and *S. aureus* (6, 12, 13). Nonetheless, coinfection with these pathogens has led to a notable surge in multidrug-resistant *Staphylococci* (14). Further clinical and molecular investigations focused on specific pathogen interactions are warranted.

This study had certain limitations. It was conducted retrospectively and is at risk of chart abstraction errors. Patient characteristics and demographics were obtained through the review of patient records, rather than through a clinical examination at the time of infection. Due to the inability to accurately track outcomes after hospital discharge, deaths that occurred outside of the hospital may not have been considered. While we utilized ID consultant notes to exclude contaminants, we acknowledge that differences in ID provider practices could lead to variability in the classification of organisms as contaminants or pathogens. Furthermore, 7% of infections lacked gene typing data, which may introduce bias and reduce the generalizability of the findings. Lastly, the low number of polymicrobial MRSA BSI cases represents a limitation of the study. Further research is required to assess the impact of polymicrobial MRSA BSI.

## MATERIALS AND METHODS

### Study design and definitions

This was a retrospective case-control study of patients with MRSA BSI admitted to Mount Sinai Hospital (MSH) in New York City between September 2014 and July 2022. Hospitalized adults aged >18 years were included if they had at least one positive blood culture for MRSA. Cases were defined as those with polymicrobial MRSA BSI, and controls were defined as those with monomicrobial MRSA BSI. Polymicrobial MRSA BSI was defined as the isolation of one or more microorganisms within 24 h before or after a positive MRSA blood culture, encompassing a 48-h timeframe. Data from the first positive blood culture event from the admission was included, aside from two individuals who developed polymicrobial BSI only during the second MRSA BSI episode. Patients were excluded if they were transferred to MSH from an outside hospital or from MSH to an outside hospital for more than 24 h after admission. Given the awareness of the contribution of SARS-CoV-2 disease status, especially since the study spanned COVID-19 pandemic, we excluded those with SARS-CoV-2 who required treatment directed at SARS-CoV-2 during the same admission for MRSA BSI, had a positive SARS-CoV-2 test within 2 weeks prior to or after the BSI diagnosis, or were symptomatic due to SARS-CoV-2 per clinical documentation. The protocol was approved by the Mount Sinai Institutional Review Board (HS #13-00981 and HS# 17-00825). Authorization for the use and disclosure of protected health information (PHI) was obtained through a waiver, and informed consent was waived. This study was performed in accordance with the Helsinki Declaration of 1964, including later amendments.

Specimen data were collected from the Clinical Microbiology Laboratory (CML) of MSH by the Mount Sinai Pathogen Surveillance Program. The CML identified organisms using matrix-assisted laser desorption/ionization time-of-flight mass spectrometry (MALDI-TOF MS) (Bruker Biotyper; Bruker Daltonics), with use of Vitek 2 (bioMérieux) and/or Microscan (Beckman Coulter) for susceptibilities according to Clinical and Laboratory Standards Institute (15) standards.

Demographic and clinical characteristics were retrospectively extracted from the electronic medical record system, and entered into a REDCap (16) database, as previously described (2), with review by at least two infectious diseases (ID) trained investigators. Particular emphasis was placed on reviewing ID notes to identify potential contaminants, defined as organisms isolated from blood culture that are not considered clinically significant (17). As it is standard practice at our institution to have ID consultations on all patients diagnosed with *S. aureus* BSI, ID clinical notes were reviewed in depth to assist in these determinations. One of the main deciding features was documentation of the ID consultant’s decision-making, especially if the ID consultants decided to provide treatment for the co-pathogens identified. Those deemed to have contaminants were added to the control group. Positive cultures on or after the fourth day following hospital admission were defined as HO MRSA, while BSI occurring during the first 72 h of hospital admission was defined as CO MRSA according to National Healthcare Safety Network definitions (18).

### Statistical analysis

For our analysis, we selected established clinical factors related to *S. aureus* infection, such as demographics, admission sources, presence of invasive devices, and sources of infection. Additionally, we examined patient outcomes and mortality, particularly those related to MRSA BSI. Differences in patient characteristics were assessed by the chi-square or Fisher exact test for categorical variables, and by the Kruskal-Wallis test for non-normal continuous variables. Given the exploratory nature of the study, the association of each risk factor on the development of polymicrobial MRSA BSI and each outcome was assessed using univariate logistic regression. Statistical significance was measured by a *P*-value < 0.05. All statistical analyses were performed using SAS Studio (19).
